# Development and Interpretation of Multiple Machine Learning Models for Predicting Postoperative Delayed Remission of Acromegaly Patients During Long-Term Follow-Up

**DOI:** 10.3389/fendo.2020.00643

**Published:** 2020-09-16

**Authors:** Congxin Dai, Yanghua Fan, Yichao Li, Xinjie Bao, Yansheng Li, Mingliang Su, Yong Yao, Kan Deng, Bing Xing, Feng Feng, Ming Feng, Renzhi Wang

**Affiliations:** ^1^Department of Neurosurgery, Peking Union Medical College Hospital, Chinese Academy of Medical Sciences and Peking Union Medical College, Beijing, China; ^2^DHC Mediway Technology Co., Ltd., Beijing, China; ^3^Department of Radiology, Peking Union Medical College Hospital, Chinese Academy of Medical Sciences and Peking Union Medical College, Beijing, China

**Keywords:** acromegaly, delayed remission, machine learning, LIME, SHAP

## Abstract

**Background:** Some patients with acromegaly do not reach the remission standard in the short term after surgery but achieve remission without additional postoperative treatment during long-term follow-up; this phenomenon is defined as postoperative delayed remission (DR). DR may complicate the interpretation of surgical outcomes in patients with acromegaly and interfere with decision-making regarding postoperative adjuvant therapy.

**Objective:** We aimed to develop and validate machine learning (ML) models for predicting DR in acromegaly patients who have not achieved remission within 6 months of surgery.

**Methods:** We enrolled 306 acromegaly patients and randomly divided them into training and test datasets. We used the recursive feature elimination (RFE) algorithm to select features and applied six ML algorithms to construct DR prediction models. The performance of these ML models was validated using receiver operating characteristics analysis. We used permutation importance, SHapley Additive exPlanations (SHAP), and local interpretable model–agnostic explanation (LIME) algorithms to determine the importance of the selected features and interpret the ML models.

**Results:** Fifty-five (17.97%) acromegaly patients met the criteria for DR, and five features (post-1w rGH, post-1w nGH, post-6m rGH, post-6m IGF-1, and post-6m nGH) were significantly associated with DR in both the training and the test datasets. After the RFE feature selection, the XGboost model, which comprised the 15 important features, had the greatest discriminatory ability (area under the curve = 0.8349, sensitivity = 0.8889, Youden's index = 0.6842). The XGboost model showed good discrimination ability and provided significantly better estimates of DR of patients with acromegaly compared with using only the Knosp grade. The results obtained from permutation importance, SHAP, and LIME algorithms showed that post-6m IGF-1 is the most important feature in XGboost algorithm prediction and showed the reliability and the clinical practicability of the XGboost model in DR prediction.

**Conclusions:** ML-based models can serve as an effective non-invasive approach to predicting DR and could aid in determining individual treatment and follow-up strategies for acromegaly patients who have not achieved remission within 6 months of surgery.

## Introduction

Acromegaly is a chronic endocrine disease that is mostly caused by growth hormone (GH)-secreting pituitary adenomas (PAs), resulting in excessive circulating levels of insulin-like growth factor 1 (IGF1) and in high morbidity and mortality (1, 2). According to the current Endocrine Society Clinical Practice Guidelines on acromegaly, transsphenoidal surgery (TSS) is the first-line treatment, and its initial cure rate for macroadenomas is 40–50% (3). The remission of acromegaly needs to meet the following two conditions at least 12 weeks after surgery: normalized levels of IGF1 and a random GH level of <1.0 μg/L or a nadir GH level of <0.4 μg/L following an oral glucose tolerance test (OGTT) (3, 4).

According to the literature and our clinical experience, some patients with acromegaly do not reach the remission standard in the short term after surgery but achieve remission without additional postoperative treatment during long-term follow-up; this phenomenon is defined as postoperative delayed remission (5, 6). Changes in GH and IGF1 levels may be inconsistent following surgery, and the reason for delayed remission may be a longer-than-expected period required for IGF1 levels to return to normal (7). The reason may also be that the residual tumor cells gradually necrotize with ischemia after surgery.

Delayed remission may affect a doctor's ability to judge the surgical response and to determine whether the patient needs postoperative adjuvant therapy. Previous studies have focused on the retrospective analysis of clinical risk factors and their associations with delayed remission, and the results have revealed that postoperative 3-month IGF1 (post-3m IGF1) levels might have a significant influence on delayed remission (6). However, the two previous studies (5, 6) on delayed remission in patients with acromegaly have used 3 months as the observation time for postoperative remission, which was too short. It is more reasonable to observe the remission of acromegaly patients within 6 months after surgery. Moreover, the prognosis should not be determined by only one feature. The combined analysis of multiple features may be more helpful for clinical treatment decision-making (8, 9). Thus, compared with a simple analysis of prognosis-related risk factors, it is more conducive to clinical use to build a prediction model with multiple important clinical features. As far as we know, there have been no previous attempts to construct a prediction model for delayed remission of acromegaly with multiple clinical features. Therefore, the establishment of a more comprehensive, effective, and widely used delayed remission prediction model has important implications for the treatment of acromegaly patients who have not achieved remission within 6 months of surgery.

Machine learning (ML) is a subset of artificial intelligence whereby knowledge and information are automatically acquired by extracting patterns from large databases (10, 11). ML is increasingly used in the medical community, particularly in the field of oncology. Previous studies have demonstrated that ML models can provide better accuracy and discrimination for the prediction of prognoses for lung adenocarcinoma (12) and breast cancer (13), chemoradiation therapy response in rectal cancer (14), radiotherapy response for acromegaly (15), surgical outcomes for head and neck cancer (16), and diagnosis for leukemia (17). For sellar region tumors, ML could be more effective for predicting a patient's clinical outcome and could provide better clinical decision support for neuroendocrinologists and neurosurgeons (18).

However, to the best of our knowledge, there have been no previous attempts to use ML algorithms to predict long-term outcomes in patients with acromegaly. Hence, the aims of the present study were to establish an ML model for predicting delayed remission and to try to explain and evaluate the interpretability of that ML model, with a view to assist in the decision-making process regarding acromegaly patients who have not achieved remission within 6 months of surgery.

## Materials and Methods

### Study Population

The present study was conducted with the participation of acromegaly patients admitted to the Department of Neurosurgery at the Peking Union Medical College Hospital (PUMCH) between January 2000 and October 2017. As shown in the Endocrine Society Clinical Practice Guideline on acromegaly (3), the preoperative diagnostic criteria for acromegaly are as follows: (1) adult patients with clinical symptoms of acromegaly (3), (2) PA confirmed by pituitary magnetic resonance imaging (MRI), and (3) preoperative IGF1 (pre-IGF1) values exceeding the upper limit of the age- and the gender-related reference range (19) and lack of suppression of GH to <1.0 ng/ml following documented hyperglycemia during an oral glucose load.

The inclusion criteria were as follows: (1) the acromegaly patients had undergone initial TSS conducted by the same experienced surgeons in the pituitary treatment group using a microscope or an endoscope in our hospital, (2) PAs had been confirmed by postoperative pathological examination, (3) at 6 months following surgery, the patients who did not meet the postoperative endocrine remission criteria [i.e., either postoperative random GH (post-rGH) levels <1.0 ng/ml or postoperative nadir GH (post-nGH) levels <0.4 ng/ml that were associated with normal age- and gender-matched IGF1 levels] (3, 4), (4) no history of radiotherapy or medical therapy following TSS, and (5) the patients had endocrine follow-up data for more than 18 months following TSS.

After screening, a total of 306 acromegaly patients were eligible for inclusion in the study. They were randomly divided into a training dataset (*n* = 244), which was used for model construction, and a test dataset (*n* = 62), which was used for model validation (i.e., a 4:1 ratio, respectively). This study was approved by the ethical review committee of the PUMCH, and the need for patients' informed consent was waived.

### Clinical Features

The following 18 relevant clinical features were collected: age, gender, tumor size, Knosp grade (20), hypertension, fasting blood glucose level, pre-rGH level, pre-IGF1 level, preoperative nadir GH (pre-nGH) level, tumor texture, cavernous sinus invasion, post-1w rGH level, post-1w IGF1 level, post-1w nGH level, Ki-67 level (<3 or ≥3%), post-6m rGH level, post-6m IGF1 level, and post-6m nGH level. The tumor size and the Knosp grade were determined using preoperative pituitary contrast-enhanced MRI images (20, 21). The cavernous sinus invasion (22) and the tumor texture (2) were determined by the surgeon during the operation. The cavernous sinus invasion of tumors was considered to be positive if the tumor extended the cavernous sinus and a cavernous sinus defect was observed (23). Tumor that could be suctioned out using an aspirator was considered as soft, while a tumor that could not be suctioned out was considered as firm (2). The Ki-67 index was defined by an immunohistochemistry assay. The definition of delayed remission is that the acromegaly patients do not meet the aforementioned endocrine remission criteria within 6 months of surgery but achieve remission during long-term follow-up (at least 18 months after surgery) without additional postoperative treatment (5). The Pearson correlation coefficient matrix between 18 clinical risk features and remission outcomes is shown in Supplementary Figure 1.

### Study Design and ML Algorithms

Before developing the ML prediction model based on the 18 clinical features mentioned above, we first supplemented the missing values according to the k-nearest neighbor algorithm (11, 24). The absence of clinical features cannot exceed 8%, and patients with more than one missing value would be excluded. The continuous data were normalized by z-score normalization (25), and the categorical data were transformed *via* one-hot encoding (26). To address the serious imbalance in the number of patients with delayed and non-delayed remission, we intend to synthesize new patient samples of delayed remission using three commonly used resampling techniques in the training dataset: the synthetic minority oversampling technique (SMOTE), SMOTETomek, and SMOTEENN (27, 28). After data resampling, the resampling technology used in the present study was determined based on the specificity value in the ML algorithm described below.

We used the following six representative supervised ML algorithms for clinical feature screening and model construction in the training dataset: logistic regression (LR), gradient boosting decision tree (GBDT), adaptive boosting (AdaBoost), extreme gradient boost (XGBoost), categorical boosting (CatBoost), and random forest (RF) (23, 29). The detailed parameters of the six algorithms are presented in Supplementary Table 1.

### Feature Selection and Model Construction

The ML predictive models for delayed remission were developed using the six algorithms on all included variables. We carried out feature selection to remove invalid features containing irrelevant or redundant information. The importance of each feature was assessed using the recursive feature elimination (RFE) algorithm, with all features being sorted according to their level of importance. After the features had been sequentially reduced in order of importance, the remaining features were introduced into the corresponding ML algorithm. We calculated the receiver operating characteristic (ROC) curves and the area under ROC (AUC) values of models with different numbers of variables. For each iteration, a random 5-fold cross-validation was performed for training dataset based on the corresponding number of clinical features. The experiment was repeated five times, and we used a grid search approach to identify the optimal parameters for each model in the training dataset (23).

We assessed the predictive performance according to the AUC, accuracy (ACC), Youden's index, and other measurement indicators (30). By comparing the AUC values of the models in the training dataset, we determined the model with the best predictive performance and externally verified it in the test dataset. DeLong test was used to compare the prediction performance of the best ML model and the Knosp grade.

### Model Interpretation

ML models usually have distinctive black box and uninterpretable characteristics, which means that the function between the features and the response is invisible to the researcher (23, 31–33).

Permutation importance is an algorithm that calculates the importance score of each feature variable of the dataset (34). The permutation feature importance is defined as the decrease in a model score when a single feature value is randomly shuffled (35). This process breaks the relationship between features and goals, so the decline in model scores indicates how much the model depends on the feature. This technique benefits from the agnostic nature of the model and can be calculated multiple times with different permutations of features. We used this widely adopted method to calculate feature importance in our ML model.

We then introduced an explanation technique called local interpretable model–agnostic explanation (LIME) (36), which explains the predictions of any classifier in an interpretable and faithful manner by learning an interpretable model locally around the prediction. Intuitively, an explanation is a local linear approximation of the model's behavior. It is more straightforward to approximate it around the vicinity of a particular instance when the model is seen as a black box. LIME perturbs the instance that used to be explained and learns a sparse linear model around it as an explanation.

The SHapley Additive exPlanations (SHAP) approach is an extension of LIME; feature weights are represented as SHapley values from game theory. The SHAP approach has a high potential for rationalizing the predictions made by complex ML models (37). In the present study, we used the SHAP method to observe the influence of each feature on the prediction results during the prediction process applied to each sample.

Finally, we used a partial correlation plot (PDP) to show the marginal effects of the most important features of the prediction results from the best ML model (38). A PDP can show whether the relationship between the target and a feature is linear, monotonic, or more complex.

### Statistical Analysis

We used version 2.7 of the Python Programming Language (Python Software Foundation, Wilmington, DE, USA) to develop and evaluate these ML models. Independent-sample *t*-tests were used to compare the differences in normal continuous features and the performance of the different ML models, and Wilcoxon test was used for non-normal continuous features.

## Results

### Patient Characteristics

After screening, 306 acromegaly patients who had not achieved the remission criteria within 6 months of surgery and had more than 18 months of follow-up data were identified and included in the study. The clinical characteristics of the patients (244 patients in the training dataset and 62 patients in the test dataset) are shown in Table 1. A total of 55 (17.97%) patients met the criteria for delayed remission: 46 (18.85%) patients in the training dataset and nine (14.52%) patients in the test dataset. We detected no significant interclass differences in any of the 18 clinical features between the training dataset and the test dataset (*p* = 0.05–0.914). The results justify the use of the two datasets as training and test datasets.

**Table 1:** Patients' characteristics of the training and the test datasets.

**Characteristic**	**Total**	**Training dataset**	**Test dataset**	***P*-value**
*N*	306	244	62	
Age (mean ± *SD*, year)	37.69 ± 11.90	38.37 ± 11.35	35.03 ± 13.63	0.05
**Gender**				
Female	162	129	33	0.96
Male	144	115	29	
**Tumor Size**				
Microadenoma	54	45	9	0.469
Macroadenoma	252	199	53	
**Knosp Grade**				
Grade 0	67	57	10	0.460
Grade 1	33	27	6	
Grade 2	50	36	14	
Grade 3	99	77	22	
Grade 4	57	47	1o	
**Hypertension**				
No	199	155	44	0.272
Yes	107	89	18	
**Fasting Blood Glucose**				
Normal	119	94	25	0.234
Impaired glucose tolerance	109	92	17	
Diabetes	78	58	20	
Pre-rGH (ng/ml)	27.50 (9.05–66.00)	28.37 (9.80–67.35)	22.35 (8.20–63.20)	0.337
Pre-IGF-1 (ng/ml)	921.31 ± 277.81	918.67 ± 278.95	931.74 ± 275.30	0.741
Pre-nGH (ng/ml)	17.60 (6.10–38.83)	18.20 (6.40–39.83)	14.40 (5.20–34.78)	0.354
**Cavernous Sinus Invasion**				
No	204	166	38	0.315
Yes	102	78	24	
**Tumor Texture**				
Soft	239	195	44	0.128
Firm	67	49	18	
**Ki-67 (%)**				
<3%	211	169	42	0.817
≥3%	95	75	20	
Post-1w rGH (ng/ml)	3.99 (1.70–10.45)	4.20 (1.70–10.95)	3.40 (1.90–9.79)	0.845
Post-1w IGF-1 (ng/ml)	701.50 (559.25–908.00)	693.00 (554.75–893.5)	730.33 (561.78–969.25)	0.367
Post-1w nGH (ng/ml)	2.80 (1.10–6.65)	2.87 (1.09–6.74)	2.39 (1.14–6.90)	0.914
Post-6m rGH (ng/ml)	3.45 (1.80–8.10)	3.50 (1.65–7.95)	3.40 (1.98–8.60)	0.772
Post-6m IGF-1 (ng/ml)	524.50 (367.00–732.25)	535.70 (367.25–739.50)	499.00 (364.50–726.25)	0.772
Post-6m nGH (ng/ml)	1.99 (0.90–4.56)	1.99 (0.90–4.37)	1.99 (0.97–5.74)	0.541
**Delayed Remission**				
No	251	198	53	0.427
Yes	55	46	9	

*SD, standard deviation; MTD, maximal tumor diameter; pre-, preoperative; rGH, random GH; nGH, nadir GH; post-1w, postoperative 1 week; post-6m, postoperative 6 months*.

*Continuous features consistent with a normal distribution were presented as mean ± standard deviation; otherwise, the median and the quartile are used. Chi-square or Fisher's exact test was used to compare the differences in categorical features*.

As shown in Table 2, both in the training and the test datasets, five features (post-1w rGH, post-1w nGH, post-6m rGH, post-6m IGF-1, and post-6m nGH) were significantly associated with the delayed remission of acromegaly patients (*p* = 0.000–0.049). Moreover, age, tumor size, Knosp grade, hypertension, pre-rGH, pre-nGH, cavernous sinus invasion, and Ki-67 index only showed a significant relationship with delayed remission in the training dataset, but there was no statistical difference in the validation dataset. However, we found no significant differences in gender, fasting blood glucose, pre-IGF-1, or tumor texture between the delayed remission and non-delayed remission groups in both the training and the test datasets (*p* = 0.057–0.454).

**Table 2:** Univariate analysis of the clinical characteristics of patients in the training and the test datasets.

**Characteristic**	**Training dataset (*****n*** **=** **244)**		***P*-value**	**Test dataset (*****n*** **=** **62)**		***P*-value**
	**Non-remission**	**Delayed remission**		**Non-remission**	**Delayed remission**	
*N*						
Age (mean ± *SD*, year)	37.62 ± 10.78	41.57 ± 13.19	0.033	35.17 ± 13.77	34.22 ± 13.56	0.849
**Gender**						
Female	108	21	0.276	29	4	0.568
Male	90	25		24	5	
**Tumor Size**						
Microadenoma	29	16	0.002	8	1	0.754
Macroadenoma	169	30		45	8	
**Knosp Grade**						
Grade 0	38	19	0.000	8	2	0.453
Grade 1	17	10		4	2	
Grade 2	28	8		12	2	
Grade 3	71	6		19	3	
Grade 4	44	3		10	0	
**Hypertension**						
No	132	23	0.034	39	5	0.271
Yes	66	23		14	4	
**Fasting Blood Glucose**						
Normal	78	16	0.454	22	3	0.876
Impaired glucose tolerance	71	21		14	3	
Diabetes	49	9		17	3	
Pre-rGH (ng/ml)	33.60 (12.07–77.55)	11.06 (4.48–30.70)	0.001	22.60 (8.20–65.65)	20.00 (10.35–26.35)	0.589
Pre-IGF-1 (ng/ml)	939.14 ± 253.71	830.52 ± 358.63	0.057	922.95 ± 274.32	983.56 ± 291.94	0.546
Pre-nGH (ng/ml)	20.45 (8.38–43.78)	9.43 (2.21–21.60)	0.001	14.40 (5.20–37.55)	14.40 (8.65–21.80)	0.920
**Cavernous Sinus Invasion**						
No	129	37	0.045	31	7	0.272
Yes	69	9		22	2	
**Tumor Texture**						
Soft	156	39	0.361	36	8	0.200
Firm	42	7		17	1	
**Ki-67 (%)**						
<3%	130	39	0.011	36	6	0.941
≥3%	68	7		17	3	
Post-1w rGH (ng/ml)	5.39 (2.18–14.85)	1.29 (0.78–2.23)	0.001	4.33 (2.30–10.10)	1.30 (1.10–2.65)	0.004
Post-1w IGF-1 (ng/ml)	756.81 ± 254.28	611.20 ± 251.13	0.001	738.66 (562.68–977.00)	704.00 (471.50–939.00)	0.478
Post-1w nGH (ng/ml)	3.91 (1.61–8.68)	0.83 (0.46–1.63)	0.000	2.92 (1.41–7.82)	1.06 (0.93–2.27)	0.033
Post-6m rGH (ng/ml)	4.50 (2.58–9.50)	1.05 (0.275–2.15)	0.000	3.70 (2.39–10.25)	0.90 (0.45–4.20)	0.033
Post-6m IGF-1 (ng/ml)	628.28 ± 248.67	332.63 ± 87.07	0.000	529.00 (379.00–782.00)	384.00 (286.00–391.50)	0.011
Post-6m nGH (ng/ml)	2.57 (1.33–5.09)	0.39 (0.12–1.02)	0.000	2.10 (1.33–6.45)	0.71 (0.24–2.41)	0.009

*SD, standard deviation; MTD, maximal tumor diameter; pre-, preoperative; rGH, random GH; nGH, nadir GH; post-1w, postoperative 1 week; post-6m, postoperative 6 months*.

*Continuous features consistent with a normal distribution were presented as mean ± standard deviation; otherwise, the median and the quartile are used. Chi-square or Fisher's exact test was used to compare the differences in categorical features*.

### Patient Resampling, Feature Selection, and Model Construction

The prediction model we build is geared to identify as many patients with acromegaly as possible with delayed remission, so the sensitivity of the model is particularly important. The evaluation of three resampling methods in six ML algorithms revealed that the SMOTEENN method had the highest sensitivity values in all six ML models (Table 3). Therefore, we chose the SMOTEENN algorithm as the most suitable resampling method for the training dataset in the present study because it was less susceptible to overfitting and had a higher prediction performance than the other resampling methods.

**Table 3:** The performance of multiple resampling methods on the six ML models.

**Resampling methods**	**SP of ML LR**	**Adaboost**	**GBDT**	**XGBoost**	**CatBoost**	**RF**
None	0.4444	0.6667	0.5556	0.6667	0.5556	0.4445
SMOTE	0.5556	0.6667	0.5556	0.5556	0.5556	0.5556
SMOTETomek	0.5556	0.6667	0.5556	0.5556	0.5556	0.4445
SMOTEENN	0.5556	0.6667	0.6667	0.7778	0.6667	0.5556

*SP, specificity; ML, machine learning; LR, logistic regression; RF, random forest*.

**Figure 1:**
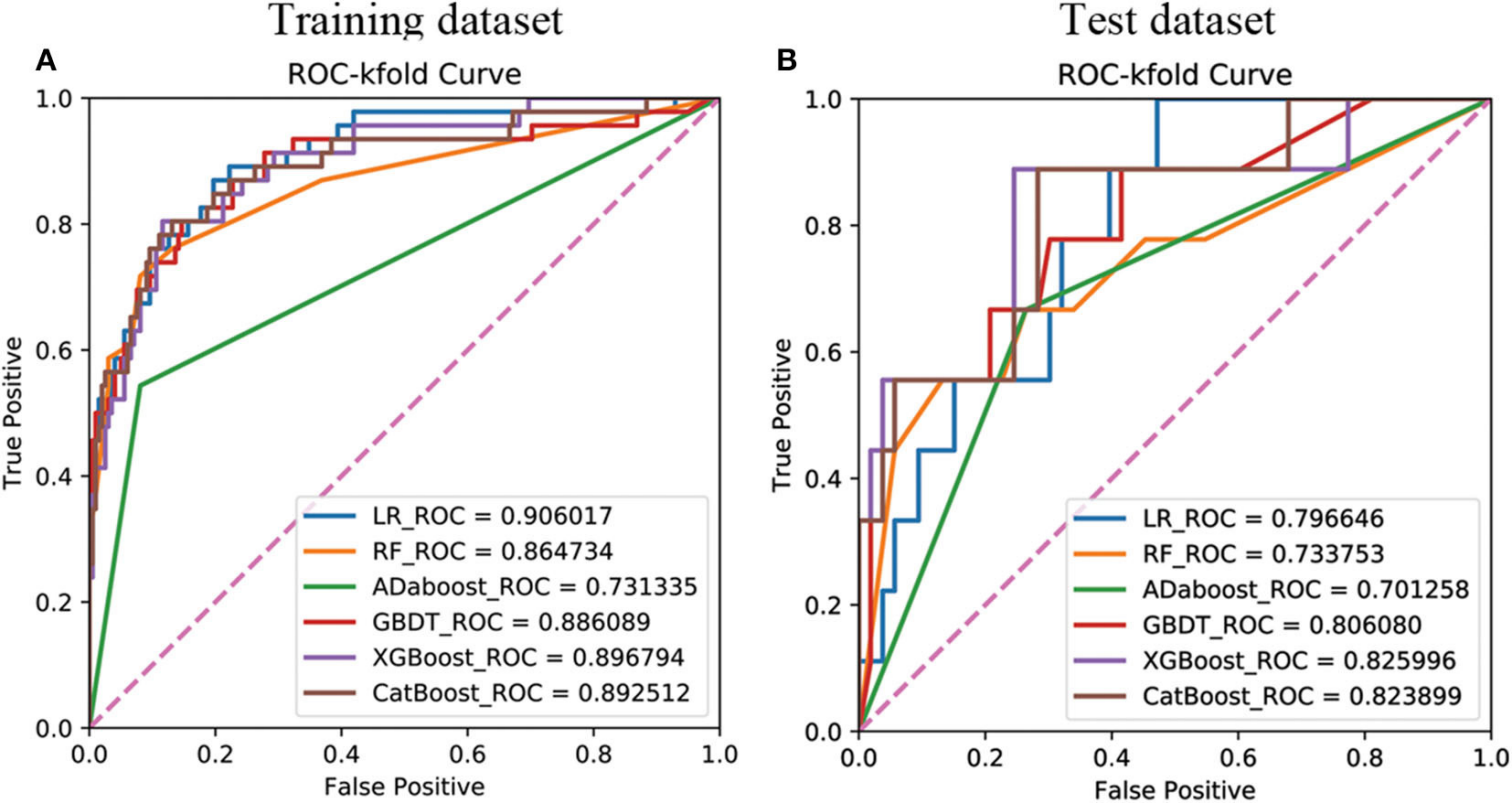
Receiver operating characteristic curves showing the delayed remission predictive performance of six machine learning algorithms based on the selected significant features in the training **(A)** and test **(B)** datasets. LR, logistic regression; GBDT, gradient boosting decision tree; XGBoost, extreme gradient boost; AdaBoost, adaptive boosting; CatBoost, categorical boosting.

The 18 available features in the training dataset were used to build delayed remission prediction models based on six ML algorithms. Through the process of RFE feature selection, we determined the optimal feature numbers and AUCs of each algorithm in the training dataset. The best predictive performance was observed in LR (AUC = 0.9060), followed by XGBoost (AUC = 0.8968), CatBoost (AUC = 0.8925), GBDT (AUC = 0.8861), RF (AUC = 0.8647), and AdaBoost (AUC = 0.7313) in the training dataset (Figure 1A).

We then verified the performance of these models in the test dataset and the AUC, ACC, sensitivity, and specificity of each ML model in the test dataset, as shown in Table 4. The results revealed that the prediction model with the highest AUC, sensitivity, and Youden's index was the XGboost model, based on the top 15 important clinical features (AUC = 0.8349, sensitivity = 0.8889, Youden's index = 0.6842) in the test dataset. However, we observed the highest values of ACC (0.7903) and specificity (0.8302) when the LR model contained all 18 clinical features in the test dataset (Figure 1B).

**Table 4:** The best performance of the six ML algorithms in the test dataset.

**Algorithms**	**LR**	**Adaboost**	**GBDT**	**XGboost**	**Catboost**	**RF**
Feature number	18	14	7	15	15	9
AUC	0.7945	0.7013	0.8061	0.8260	0.8239	0.7338
Threshold	0.00008	1	0.9997	0.6041	0.2743	0.9
Youden index	0.3858	0.4025	0.4759	0.6436	0.4025	0.3292
ACC	0.7903	0.7258	0.7097	0.7742	0.7258	0.7419
Specificity	0.8302	0.7358	0.6981	0.7547	0.7358	0.7736
Sensitivity	0.5556	0.6667	0.7778	0.8889	0.6667	0.5556
PPV	0.3571	0.3	0.3043	0.381	0.3	0.2941
NPV	0.9167	0.9286	0.9487	0.9756	0.9286	0.9111
PLR	3.2716	2.5238	2.5764	3.6239	2.5238	2.4537
NLR	0.5354	0.453	0.3183	0.1472	0.453	0.5745

*LR, logistic regression; RF, random forest; AUC, area under the curve; ACC, accuracy; PPV, positive predictive value; NPV, negative predictive value; PLR, positive likelihood ratio; NLR, negative likelihood ratio*.

The results of the DeLong test suggested that the prediction performance of the XGboost model was significantly better than that of using only the Knosp grade in the training dataset (AUC = 0.7130) and the test dataset (AUC = 0.665). Finally, as described above, according to the best sensitivity, we choose XGboost model as our final prediction model.

### Feature Importance

**Figure 2:**
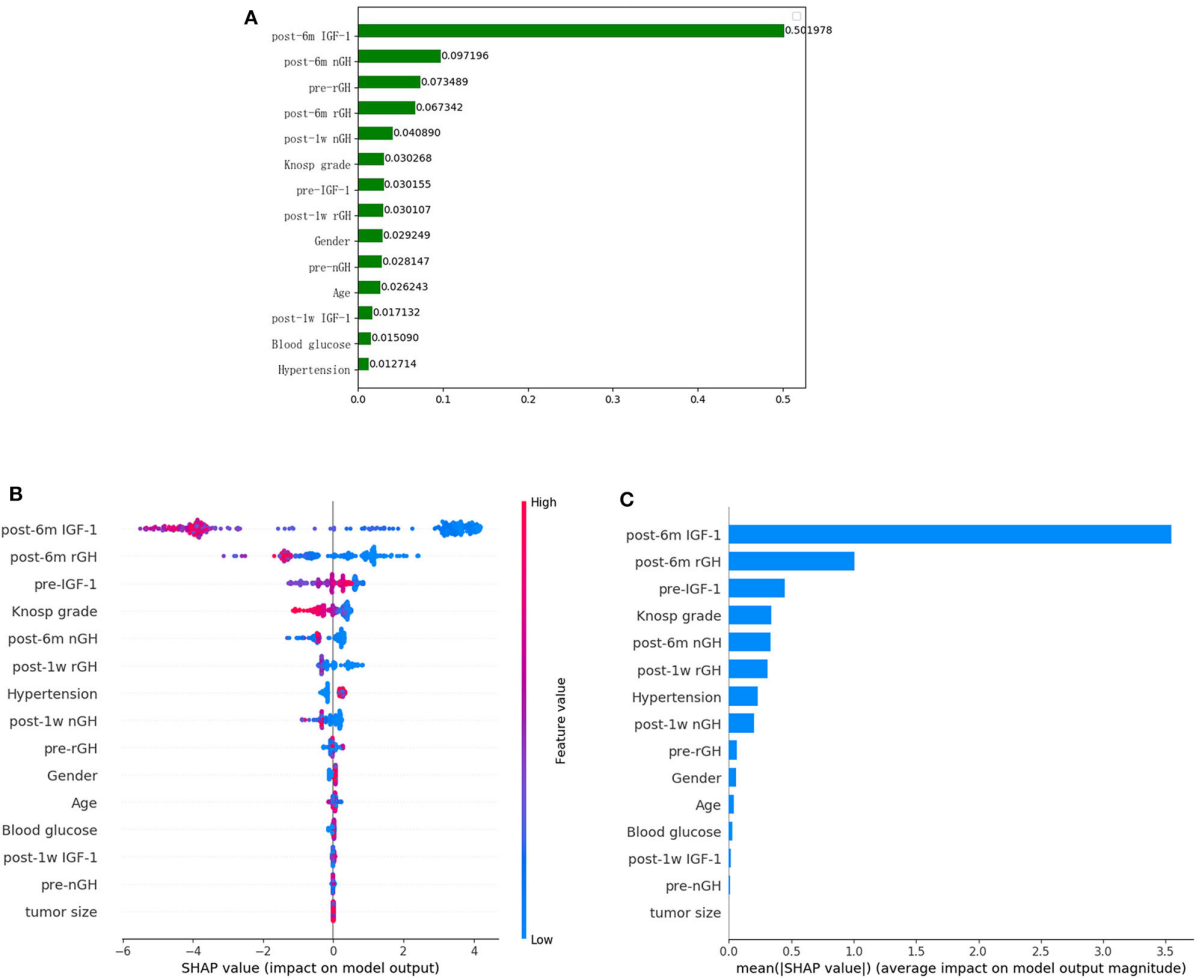
Feature importance ranking based on permutation importance **(A)** and SHapley Additive exPlanations (SHAP) values **(B,C)** in XGboost model. **(A)** The features are ranked based on the permutation importance method in the XGboost model. **(B)** The features are ranked according to the sum of the SHAP values for all patients, and the SHAP values are used to show the distribution of the effect of each feature on the XGboost model outputs. Red indicates that the value of a feature is high, and blue indicates that the value of a feature is low. The x-axis indicates the effect of SHAP values on the model output. The larger the value of the x-axis, the greater the probability of delayed remission. **(C)** Standard bar charts were drawn and sorted using the average absolute value of the shape values of each feature in the XGboost model.

After the application of the classifier-specific feature evaluator for the XGboost model, the included features were ranked based on their information gain; the results of permutation importance demonstrated that the top two risk features were post-6m IGF-1 and post-6m nGH (Figure 2A).

To further understand and get an overview on the importance of the features, we implemented the SHAP algorithm, which can identify and map clinical features to the molecular graphs by increasing or decreasing the probability of the predicted activities, thereby enabling the visualization of structural patterns that determine predictions. The top two risk features were post-6m IGF1 and post-6m rGH, as shown in Figures 2B, C; the lower the values of the two features, the more likely the chance of delayed remission.

Univariate and multivariate logistic regression analysis was used to determine the independent clinical risk variables for delayed remission. Similar to the previous results of SHAP, we found a significant association between delayed remission and post-6m IGF1 (OR = 0.991, 95% CI 0.987–0.995, *p* = 0.000), which means that high post-6m IGF1 tends to achieve a lower delayed remission ratio. Another significant predictor is post-6m nGH; a lower post-6m rGH value is linked to a higher delayed remission ratio (OR = 0.615, 95% CI 0.437–0.866, *p* = 0.005) (Table 5).

**Table 5:** Univariate and multivariate analyses measure the correlation between the clinical features and the delayed remission.

**Variable**	**Univariate analysis**			**Multivariate analysis**		
	**Odds ratio (OR)**	**95% CI**	***p*-value**	**OR**	**95% CI**	***p*-value**
Age	1.023	0.998–1.049	0.067			
Gender	1.442	0.803–2.591	0.221			
Tumor size	0.386	0.198–0.755	0.005	0.539	0.189–1.535	0.247
Knosp grade	0.592	0.476–0.736	0.000	0.725	0.522–1.029	0.072
Hypertension	2.061	1.141–3.724	0.017	1.674	0.714–3.925	0.236
Fasting blood glucose	1.013	0.701–1.465	0.945			
Pre-rGH (ng/ml)	0.991	0.984–0.998	0.018	1.005	0.974–1.037	0.771
Pre-IGF-1(ng/ml)	0.999	0.998–1.000	0.054			
Pre-nGH (ng/ml)	0.989	0.978–0.999	0.036	1.016	0.974–1.037	0.473
Cavernous sinus invasion	0.44	0.216–0.893	0.023	0.814	0.272–2.438	0.713
Tumor texture	0.554	0.248–1.238	0.150			
Ki-67 (%)	0.434	0.208–0.904	0.026	0.605	0.213–1.720	0.346
Post-1w rGH (ng/ml)	0.917	0.863–0.973	0.004	1.031	0.839–1.267	0.771
Post-1w IGF-1 (ng/ml)	0.998	0.996–0.999	0.001	1.000	0.998–1.002	0.661
Post-1w nGH (ng/ml)	0.903	0.837–0.974	0.008	1.003	0.762–1.320	0.985
Post-6m rGH (ng/ml)	0.488	0.379–0.627	0.000	0.615	0.437–0.866	0.005
Post-6m IGF-1 (ng/ml)	0.991	0.988–0.994	0.000	0.991	0.987–0.995	0.000
Post-6m nGH (ng/ml)	0.249	0.155–0.400	0.000	0.54	0.285–1.022	0.058

### Model Interpretation

**Figure 3:**
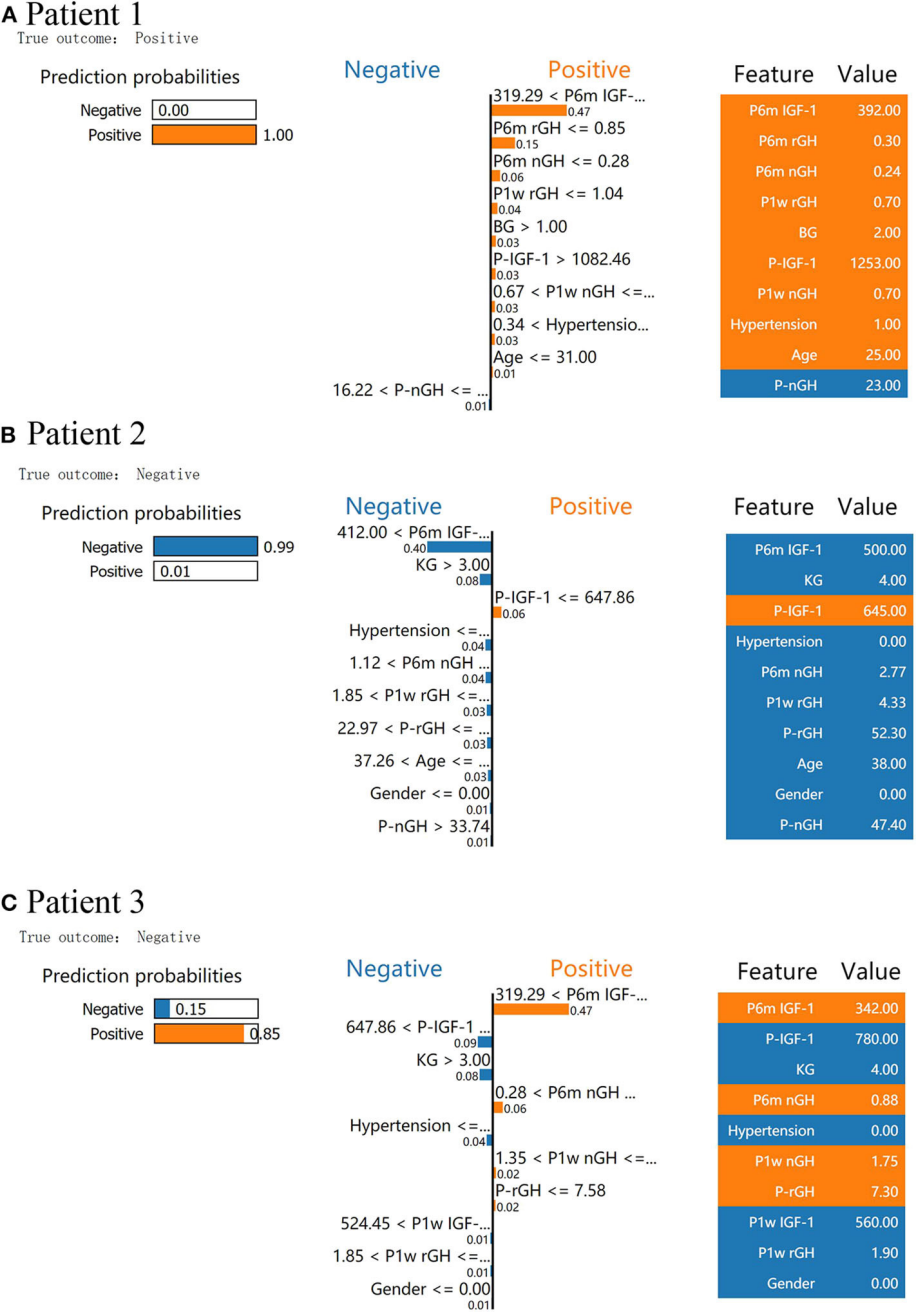
Results of local interpretable model–agnostic explanation (LIME) with XGBoost classifiers applied to two correctly predicted patients [one negative (non-delayed remission) and one positive (delayed remission) patient)] and one incorrectly predicted patient (non-delayed remission patient, incorrectly predicted with high probabilities of delayed remission). The figure reveals the role of various features in the incidence of delayed remission in each patient. The first column represents the prediction probabilities of negative and positive results achieved from the classifiers. The second column shows the contributions made by the features included in the models to the probability. The third column displays the original data values of these features. **(A)** LIME explanation for patient 1 as true positive, **(B)** LIME explanation for patient 2 as true negative, and **(C)** LIME explanation for patient 3 as false positive.

We used LIME to investigate the feature contributions of each prediction. First, in the test dataset, we presented two patients that had been correctly predicted by the XGBoost prediction models. Usually, the interpretations generated by correctly predicted patients are intuitive and clear: patient 1 from the “true positive” group was correctly predicted as having a high probability of delayed remission (Figure 3A), and patient 2 from the “true negative” group was correctly predicted as having a low probability of delayed remission (Figure 3B).

In Figure 3A, XGboost predicts a 100% probability of delayed remission in patient 1, and the prediction is mainly based on post-6m IGF1 = 392.00 ng/ml, post-6m rGH = 0.3 < 0.85 ng/ml, and post-3m nGH = 0.24 < 0.28 ng/ml. We found delayed remission in patient 1 during follow-up, and the XGBoost model accurately predicted delayed remission in patient 1. After follow-up, patient 2 did not achieve delayed remission, and XGBoost predicted a 99% probability of non-delayed remission (Figure 3B). According to the data for patient 2, post-6m IGF1 = 500.00 > 412.00 ng/ml, Knosp grade = 4 > 3, and post-6m nGH > 1.12, which contribute to the negative prediction.

An understanding of the reason behind the incorrect interpretation of the model prediction will increase the clinicians' trust in model behavior and performance. After checking, the XGboost model was correct in predicting all patients with delayed remission in the test dataset. Therefore, we presented a patient 3 with “false positive” predictions (non-delayed remission patient, incorrectly predicted with high probabilities of delayed remission) by the XGBoost model (Figure 3C). The results showed that post-6m IGF1, post-6m nGH, post-1w nGH, and pre-1rGH were the most influential features that caused the prediction error in the XGboost model.

### Partial Correlation Plot

**Figure 4:**
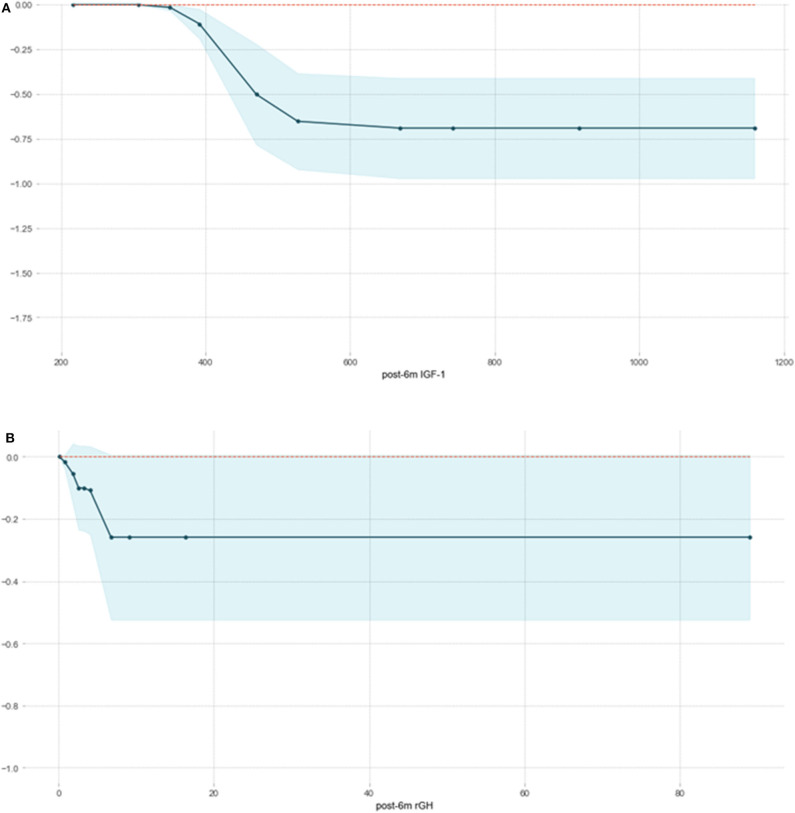
Partial correlation plot of delayed remission probability based on post-6m IGF1 **(A)** and post-6m rGH **(B)** in the XGBoost model. The y-axis represents the predicted probability compared with the baseline, and the x-axis represents the value of post-6m IGF1or post-6m rGH. The blue areas represent confidence intervals.

We fitted an XGBoost model to predict delayed remission and used PDP to visualize the relationships learned by the model. The influence and the marginal effect of post-6m IGF1 and post-6m rGH—the two most important features of the model—on the predicted delayed remission are presented in Figure 4. The results showed that, as the values continued to increase, the effect of post-6m IGF1 and post-6m rGH on the model gradually increased: the higher the value of post-6m IGF1 or post-6m rGH, the lower the delayed remission probability. However, when the value of post-6m IGF1increased above 510 ng/ml (Figure 4A) or the value of post-6m rGH increased above 7.0 ng/ml (Figure 4B), the effect tended to remain constant. These results make sense in the context of the clinical prediction of delayed remission and support the reliability of our prediction models.

## Discussion

In the present study, we developed and validated six ML models for predicting whether acromegaly patients who had not achieved remission in 6 months after TSS would experience delayed remission in long-term follow-up. The XGboost model demonstrated favorable performance as an effective non-invasive tool for determining individual treatment strategies for acromegaly patients.

As already mentioned, according to the current endocrine guidelines, it is customary to judge a patient's surgical response on whether they will achieve endocrine remission within at least 3 months after surgery (3). Patients who have not been cured by surgery usually require further postoperative treatment to control the symptoms and the progression of acromegaly (39). However, some acromegaly patients experience delayed remission without adjuvant postoperative therapy during long-term follow-up (5). The underlying mechanism of delayed remission in acromegaly after TSS remains unclear. One possible hypothesis for delayed remission is that it takes longer than expected for IGF1 levels to return to normal (7). Another hypothesis is that there are still some residual GH-secreting tumor cells after pituitary adenoma resection. Although the GH level is decreased after the operation, it is still higher than the normal range, so the patients cannot reach the remission standard in the short time after the operation. However, because the previous operation destroyed the blood supply of tumor cells, resulting in tumor cell ischemia and necrosis, the secretion level of GH gradually decreased, and then these patients eventually found in long-term follow-up that delayed remission was achieved without postoperative adjuvant treatment (5). Delayed remission may affect a doctor's ability to judge the surgical response and to determine whether the patient requires postoperative adjuvant therapy. Therefore, the accurate identification of delayed remission in short-term “unremission” acromegaly patients can be helpful with regard to decisions on long-term follow-up and treatment strategies.

Previous studies have focused on the retrospective analysis of clinical risk factors and their associations with delayed remission. Wang et al. found that the values of Knosp grade, post-1w rGH, post-1w nGH, post-3m rGH, post-3m IGF1, and post-3m nGH differed significantly between a delayed remission group and a persistent non-remission group (5). Shen et al. found that post-3m IGF1 can be used as a predictor of delayed remission in long-term follow-up (6). The two studies (5, 6) used 3 months as the observation time for postoperative remission, which was too short. Moreover, it is generally believed that a prognosis should not be determined by only one risk factor and that the combined analysis of multiple features is more valuable (40). To date, many studies have demonstrated that the ML approach provides more accurate predictive power than conventional methods with regard to the diagnosis, treatment, and prognosis of saddle region diseases (18) and multiple tumors (12, 41, 42). However, no predictive models for delayed remission in acromegaly patients have been developed. Therefore, in the present study, we retrospectively included 306 acromegaly patients who had not met the remission criteria within 6 months of surgery and established six delayed remission ML prediction models based on 18 clinical features. The six models maintained high performance, with AUCs ranging from 0.7013 to 0.8260 and ACCs ranging from 0.7097 to 0.7903 in the test dataset. The multiple clinical risk features prediction model with the highest AUC, sensitivity, and Youden's index was XGboost, and the prediction performance of the XGboost model was significantly better than that of using only the Knosp grade. The XGboost model showed the best predictive performance and was determined to be the final model used for this study and for clinical use.

Our research has some advantages. First, as with the results of previous studies, the ratio of patients with delayed remission to those with persistent non-remission was 55:251, which demonstrates a significant data imbalance in our data. When performing ML on unbalanced datasets, a small number of samples may not be detected, resulting in learning failure (43). The SMOTE technique can generate a minority class within overlapping areas and is a promising method for dealing with imbalanced datasets. Previous research has demonstrated that SMOTE can also help solve the problem of dataset imbalance in the medical field, such as in the context of type 2 diabetes prediction (44) and lung nodule recognition (45). In the present study, for the patients in the minority class (the delayed remission group), the SMOTE algorithm was able to find *k* samples (usually five) closest in distance to the minority sample. The distance between the minority sample and its nearest five neighbors was obtained from the standard Euclidean distance. As demonstrated by Ramezankhani et al. (44), synthetic new samples are generated according to the variables and the distance between a minority sample and its nearest neighbor. SMOTEENN and SMOTETomek are new methods derived from SMOTE and aim to eliminate the potentially poor-quality samples generated by SMOTE (27, 28, 46). These generated patients are created based on the characteristics of the original dataset, so they are similar to the original patients in the minority class (the delayed remission group) (47). Based on the evaluation of these three resampling methods of the six ML algorithms, we confirmed that SMOTEENN was the most suitable method for the data in our study.

Second, one disadvantage of ML is that it is considered as a “black box” without a transparent interpretation of the learning process or the outputs, and the function between the clinical features and the response is invisible to the doctor (48). However, it is necessary for doctors to understand the reasons for the ML models to make such predictions in clinical settings and to provide expert knowledge-based validation for the interpretation of ML model outputs. Therefore, in the present study, we first introduced SHAP—a conceptual new agnostic interpretation method—to explain the output-delayed remission prediction ML models. Before SHAP was widely used, researchers often used feature importance or partial dependence plots to explain the ML model. However, although these methods reveal the contribution made by their features to the predictive ability of the model, it is impossible to judge whether the influence of these features on the final forecast is positive or negative. In 2017, Lundberg and Lee proposed the wide application of the SHAP method to explain various complex models (including the black box model). SHAP connects game theory with local explanations, uniting several previous methods, and representing the possible consistent and locally accurate additive feature attribution method based on expectations (49). Compared with conventional feature importance, SHAP has the following two advantages: First, it solves the problem of multicollinearity: it considers not only the influence of a single feature but also the synergy between features. Second, it clarifies whether the influence of a feature is positive or negative. In the present research, we used SHAP to explain why the XGboost model exhibited the best performance and found that the top two risk features were post-6m IGF1 and post-6m rGH; the lower the values of these two features, the greater the likelihood of delayed remission. This result is consistent with clinical cognition and the results from previous studies (5, 6) and clinical practice and further verifies the reliability of the XGboost model. It also demonstrated that the hormone level within 1 week after surgery has poor performance in predicting the long-term prognosis of patients with acromegaly, and the hormone level at 6 months after surgery can play a more important role.

Moreover, it is well-known that explaining the prediction of the black box ML model has become a key issue and is gaining momentum. In particular, achieving the best performance of ML models is not the only focus of data scientists. People are increasingly concerned about the need to explain the predictions of black-box ML models at the global and the local levels (50). Therefore, we introduced a technique called LIME (51), which explains the predictions of any classifier in an interpretable and faithful manner by learning an interpretable model locally around the prediction. Intuitively, an explanation is a local linear approximation of the model's behavior. It is more straightforward to approximate it around the vicinity of a particular instance when the model is seen as a black box. LIME perturbs the instance that used to be explained and learns a sparse linear model around it as an explanation. In the present study, we used the LIME technique to clarify the explanations produced by two correctly predicted patients and to understand the causes and the explanations of the model's incorrect prediction, which will greatly increase a clinician's trust in model behavior and performance. Finally, PDP was used to explain the marginal effects of post-6m IGF1 and post-6m rGH, the two most important features of the XGBoost model. This makes sense in the context of the clinical prediction of delayed remission and helps to confirm the reliability of our prediction models. Furthermore, compared with a simple correlation analysis between clinical factors and prognosis, our ML model has the ability to discover and integrate clinical features that are meaningful for prognosis and can give specific prognostic probability values.

The present study also has some limitations. First, this is a single-center retrospective study involving a small number of patients, so more patients from multiple sources are required to validate the robustness and the repeatability of our model. Second, prospective studies are needed to help confirm the reliability of our model. Third, the follow-up period (at least 18 months post-operation) was relatively short. Because patients who have not achieved remission for a long time after surgery usually undergo adjuvant therapy and therefore would not meet the inclusion criteria of the present study and because ML algorithms need a relatively large sample size to avoid overfitting, we decided to evaluate patients who were followed up for ≥18 months to obtain a larger sample. Finally, in future studies, clinical ML models should be combined with radiomics to build a more comprehensive and accurate predictive model.

## Conclusion

In conclusion, it is feasible to use ML-based model to predict delayed remission or persistent active disease in patients with acromegaly whose remission status is uncertain. The use of ML model containing multiple clinical features can serve as an effective non-invasive approach to predict delayed remission and could aid in determining individual treatment and follow-up strategies for acromegaly patients who have not achieved remission within 6 months of surgery.

## Data Availability Statement

The raw data supporting the conclusions of this article will be made available by the authors, without undue reservation.

## Ethics Statement

The studies involving human participants were reviewed and approved by the Ethical Review Committee of Peking Union Medical College Hospital. Written informed consent for participation was not required for this study in accordance with the national legislation and the institutional requirements.

## Author Contributions

CD, YF, and YaL revised the manuscript for important intellectual content. RW and MF take final responsibility for this article. All authors provided contributions to study conception, design, acquisition of data, analysis, interpretation of data, drafting of the article, revising it critically for important intellectual content, and final approval of the version to be published.

## Conflict of Interest

YiL, YaL, and MS were employed by the company DHC Mediway Technology Co., Ltd. The remaining authors declare that the research was conducted in the absence of any commercial or financial relationships that could be construed as a potential conflict of interest.

## Supplementary Material

Supplementary Figure 1 The Pearson correlation coefficient matrix between 18 risk characteristics and mitigation outcomes. Blue indicates a positive correlation, and red indicates a negative correlation. The darker the color, the greater the absolute value of the correlation coefficient and the closer the corresponding features are.

## Abbreviations

PAs: pituitary adenomas
IGF1: insulin-like growth factor 1
GH: growth hormone
OGTT: oral glucose tolerance test
post-3m: postoperative 3 months
ML: machine learning
MRI: magnetic resonance imaging
pre-rGH: preoperative random GH
pre-nGH: preoperative nadir GH
post-rGH: postoperative random GH
post-nGH: postoperative nadir GH
MTD: maximal tumor diameter
IHC: immunohistochemistry
KNN: k-nearest neighbor
LR: logistic regression
GBDT: gradient boosting decision tree
XGBoost: extreme gradient boost
AdaBoost: adaptive boosting
CatBoost: categorical boosting
RF: random forest
RFE: recursive feature elimination
AUC: area under curve
ACC: accuracy
PPV: positive predictive value
NPV: negative predictive value
PLR: positive likelihood ratio
NLR: negative likelihood ratio
LIME: local interpretable model–agnostic explanation
SHAP: SHapley Additive exPlanations
PDP: partial correlation plot.
