# Supplementary figures and images for: Development and Interpretation of Multiple Machine Learning Models for Predicting Postoperative Delayed Remission of Acromegaly Patients During Long-Term Follow-Up
Source: Front Endocrinol (Lausanne). 2020 Sep 16;11:643. doi: 10.3389/fendo.2020.00643 (PMC7525125; doi:10.3389/fendo.2020.00643)

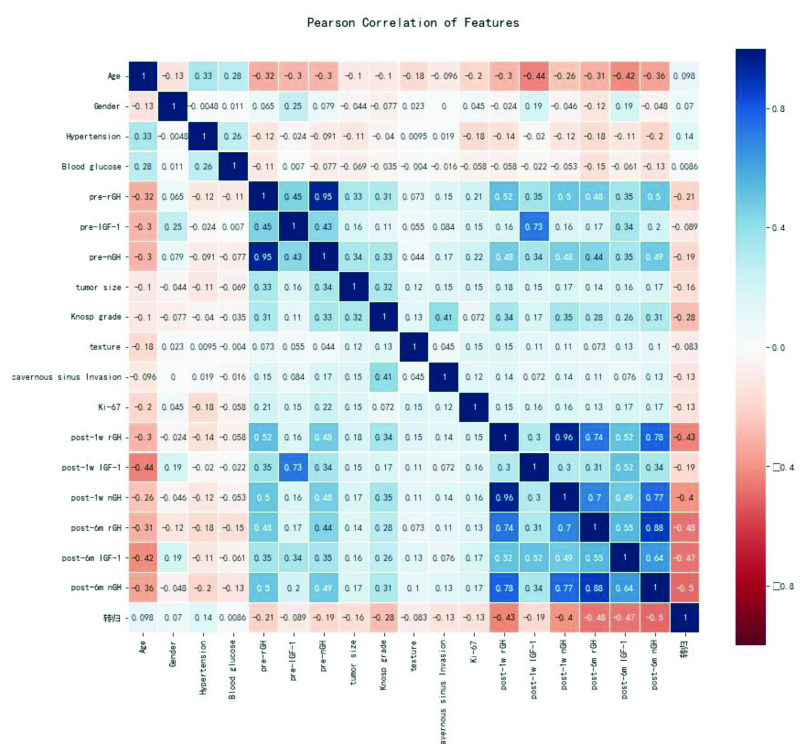

Supplement: Supplementary Figure 1 — The Pearson correlation coefficient matrix between 18 risk characteristics and mitigation outcomes. Blue indicates a positive correlation, and red indicates a negative correlation. The darker the color, the greater the absolute value of the correlation coefficient and the closer the corresponding features are. [file Image_1.TIF]
