# Supplementary material for: Development and Interpretation of Multiple Machine Learning Models for Predicting Postoperative Delayed Remission of Acromegaly Patients During Long-Term Follow-Up
Source: Front Endocrinol (Lausanne). 2020 Sep 16;11:643. doi: 10.3389/fendo.2020.00643 (PMC7525125; doi:10.3389/fendo.2020.00643)
Supplement: Supplementary file 2 [file Table_1.DOCX]

**Supplemental Table 1.** Algorithms’ parameters. This table illustrates parameter details of each algorithm.

| **Algorithms** | **Parameters** |
| --- | --- |
| Logistic regression | linear_model.LogisticRegression(C=0.1,  class_weight='balanced',dual=False, fit_intercept=True,intercept_scaling=1, l1_ratio=None,max_iter=100,multi_class='warn',  n_jobs=None,penalty='l1',random_state=None,  solver='liblinear',tol=0.0001,verbose=0,  warm_start=False) |
| Adaboost | AdaBoostClassifier(DecisionTreeClassifier(max_depth=11,min_samples_split=3,random_state=0,min_samples_leaf=16,max_features=6),learning_rate=0.2,n_estimators = 46) |
| GBDT | GradientBoostingClassifier(learning_rate=0.1, n_estimators=63,max_depth=5, min_samples_leaf =15,  min_samples_split=7,max_features=4,subsample=0.9, random_state=0) |
| XGBoost | XGBClassifier(base_score=0.5,booster='gbtree', colsample_bylevel=1,colsample_bynode=1, colsample_bytree=1,gamma=0,learning_rate=0.1, max_delta_step=0,max_depth=3,min_child_weight=1, missing=None, n_estimators=100, n_jobs=1,  nthread=None,objective='binary:logistic', params={'colsample_bytree': 0.8, 'eval_metric': 'auc', 'gamma': 0,'learning_rate': 0.5, 'max_depth': 2,  'min_child_weight':1, 'n_estimators': 20, 'nthread': 4,  'objective': 'binary:logistic', 'reg_alpha': 0.1,  'scale_pos_weight': 3, 'subsample': 0.8},  random_state=0,reg_alpha=0,reg_lambda=1,scale_pos_weight=1,seed=None, silent=None, subsample=1, verbosity=1) |
| CatBoost | CatBoostClassifier( iterations=500,  scale_pos_weight=2.5,random_seed=0,depth=3,  learning_rate= 0.1,thread_count=4,random_seed=0) |
| Random forest | RandomForestClassifier(bootstrap=True, class_weight=None,  criterion='gini', max_depth=None, max_features='auto',  max_leaf_nodes=None, min_impurity_decrease=0.0,  min_impurity_split=None, min_samples_leaf=1,  min_samples_split=2, min_weight_fraction_leaf=0.0,  n_estimators=10, n_jobs=None, oob_score=False,  random_state=0, verbose=0, warm_start=False) |
